# Biological control of potato common scab and growth promotion of potato by *Bacillus velezensis* Y6

**DOI:** 10.3389/fmicb.2023.1295107

**Published:** 2023-12-11

**Authors:** Huan Tao, Shisong Wang, Xiaoyu Li, Xiaobo Li, Jianying Cai, Lanfeng Zhao, Jia Wang, Ji Zeng, Yuzhi Qin, Xingyao Xiong, Yanfei Cai

**Affiliations:** ^1^College of Natural Resources and Environment, South China Agricultural University, Guangzhou, China; ^2^Guangdong Provincial Key Laboratory of Crop Genetic Improvement, Crops Research Institute, Guangdong Academy of Agricultural Sciences, Guangzhou, China; ^3^Guangdong Institute Center of Wine and Spirits, Guangdong Institute of Food Inspection, Guangzhou, China; ^4^Department of Pharmaceutical Engineering, School of Biomedical and Pharmaceutical Sciences, Guangdong University of Technology, Guangzhou, China; ^5^Engineering Research Center for Horticultural Crop Germplasm Creation and New Variety Breeding, Ministry of Education Changsha, Hunan Provincial Engineering Research Center for Potatoes, Southern Regional Collaborative Innovation Center for Grain and Oil Crops in China, Key Laboratory for Vegetable Biology of Hunan Province, College of Horticulture, Hunan Agricultural University, Changsha, China; ^6^Agricultural Genomics Institute at Shenzhen, Chinese Academy of Agricultural Sciences, Shenzhen, China

**Keywords:** potato, *Bacillus velezensis*, *Streptomyces scabies*, antagonism, lipopeptide, transcriptome

## Abstract

Potato common scab, caused mainly by *Streptomyces scabies*, causes surface necrosis and reduces the economic value of potato tubers, but effective chemical control is still lacking. In this study, an attempt was made to control potato common scab by inoculating potatoes with *Bacillus velezensis* (*B. velezensis*) and to further investigate the mechanism of biological control. The results showed that *B. velezensis* Y6 could reduce the disease severity of potato common scab from 49.92 ± 25.74% [inoculated with *Streptomyces scabies* (*S. scabies*) only] to 5.56 ± 1.89% (inoculated with *S. scabies* and Y6 on the same day) and increase the potato yield by 37.32% compared with the control under pot experiment in this study. Moreover, in the field trial, it was found that Y6 could also significantly reduce disease severity from 13.20 ± 1.00% to 4.00 ± 0.70% and increase the potato yield from 2.07 ± 0.10 ton/mu to 2.87 ± 0.28 ton/mu (*p* < 0.01; Tukey’s test). Furthermore, RNA-seq analysis indicated that 256 potato genes were upregulated and 183 potato genes were downregulated in response to *B. velezensis* Y6 inoculation. In addition, strain Y6 was found to induce the expression of plant growth-related genes in potato, including cell wall organization, biogenesis, brassinosteroid biosynthesis, and plant hormone transduction genes, by 1.01–4.29 times. As well as up-regulate hydroquinone metabolism-related genes and several transcription factors (bHLH, MYB, and NAC) by 1.13–4.21 times. In summary, our study will help to understand the molecular mechanism of biological control of potato common scab and improve potato yield.

## 1 Introduction

Potato (*Solanum tuberosum* L.) is the largest vegetable crop grown in a wide range of countries worldwide, it ranks fourth in the world in terms of production volume after rice, wheat, and corn (Zhang et al., 2017). It provides the world with the cheapest source of protein, carbohydrates, minerals, and vitamins (Batool et al., 2020). To ensure food security, a stable increase in potato production is required. However, Potato plants are particularly susceptible to heat, drought, soil salinity as well as parasites and pathogens such as *Streptomyces scabies* (Dahal et al., 2019). Especially, the pathogenic fungus *S. scabies* can cause potato common scab on potatoes, which is a serious soil-borne disease (Loria et al., 2006). Potato common scab can deteriorate the quality of tubers, affecting market value and causing serious economic losses. Traditional methods of scab control, such as immediate irrigation, lowering soil pH, crop rotation, and breeding for disease resistance, are often ineffective, and the use of chemical insecticides such as fludioxonil, difenacoum, fludioxonil, and chloropicrin can be effective (Santos-Cervantes et al., 2016; Braun et al., 2017; Majeed and Muhammad, 2020). However, the use of large quantities of chemical pesticides can lead to soil environmental pollution and the emergence of resistant strains of bacteria. In order to overcome this shortcoming, utilizing plant growth-promoting rhizobacteria (PGPR) to control potato common scab and promote potato production has been developed as an environmentally friendly and sustainable method in recent years (Etesami and Maheshwari, 2018).

Plant growth-promoting rhizobacteria (PGPRs) are soil bacteria that can significantly promote the growth of many plants (Di Salvo et al., 2018). PGPR can stimulate plant development via both direct and indirect processes (Santoro et al., 2015). The direct stimulation methods include nitrogen fixation (Anand et al., 2013), phosphate solubilization (Son et al., 2006), siderophore secretion, as well as release of hormones including auxin (IAA) (Lata et al., 2006), cytokinin (CTK) (Timmusk et al., 1999), and gibberellin (GA) (Bottini et al., 2004), which can stimulate root elongation and improve plant nutrient absorption (Liu et al., 2020). Indirect plant growth stimulation mechanisms involve various biocontrol mechanisms against pathogens by biosynthesizing secondary metabolites, such as cell-wall-degrading enzymes, and antibiotic lipopeptides, including surfactin, fengycin, and iturin (Cao et al., 2018). Besides, another key indirect growth-promoting function of PGPR is generating systemic resistance in host plants (Ryu et al., 2004).

*Bacillus velezensis* (*B. velezensis*), a PGPR family member, has been demonstrated to boost plant development and resilience to environmental challenges through both direct and indirect mechanisms (Yan et al., 2022). Research by Wang et al. (2022) showed that *B. velezensis* SX13 could enhance cucumber growth and output by speeding the absorption of nutrients and improving plant photosynthetic metabolism. *B. velezensis* YYC could increase the activity of defense-related enzymes, such as PAL, POD, and SOD to enhance plant basal immunity and could induce the expression of tomato genes related to auxin, gibberellin, jasmonic acid, and salicylic acid, which promote tomato growth (Yan et al., 2022). Wei et al. (2020) reported that cocultured with *B. velezens* D2WM and *B. velezensis* ZJ-1 could improve the growth and quality of *Anoectochilus* plants via promoting nutrient assimilation and increasing the abundance of beneficial microorganisms in rhizosphere soil. Jiang et al. (2019) found that *B. velezensis* was F21, which could promote the growth of watermelon and induce systemic resistance. The above studies showed that *B. velezensis* could increase plant growth and resistance to biotic and abiotic stresses under laboratory and greenhouse conditions, and possessed important research value for understanding the mechanisms of PGPR utilization to enhance plant growth and environmental stress resistance. Although there are many studies on the mechanism of interactions between PGPR and plants, there are still few studies on the mechanisms of interactions between PGPR and potato.

In this study, we investigated the effects of *B. velezensis* strain Y6 on potato via pot trials and field experiments. Then, we conducted RNA-seq of potato roots inoculated with strain Y6 under field conditions to investigate the mechanism of strain Y6 on biological control of potato common scab and growth promotion of potato. Our study will contribute to a better understanding of the molecular mechanisms of Y6 biocontrol of potato scab and enhancement of potato yield, and provide a theoretical basis for the use of *B. velezensis* strain Y6 as a specific PGPR for potato.

## 2 Materials and methods

### 2.1 Strains, plant material, and growth conditions

The strain Y6 was previously isolated from the rhizosphere soil of tomato plants in Yuejin Farm, Guangzhou, China (23° 08′N 113° 16′E) and the mutants deficient in lipopeptide (LP) synthesis were previously constructed (Cao et al., 2018), these strains were stored in our laboratory in Guangzhou, China. Strain Y6 was cultivated for no more than 9 h on Luria-Bertani (LB) liquid medium at 37°C and 180 rpm/min. It was then inoculated at 1% into new medium and incubated with shaking until OD_600_ = 1, The cell suspensions (diluted to ∼10^6^ cells per milliliter) were used for antagonism against *S. scabies* and growth promotion trials in pots. In addition, strain Y6 was produced as a biofertilizer through high-density fermentation, and the biofertilizer product was used for potato growth promotion assays in the field. The *S. scabies* was grown on YME medium (yeast extract 4 g/L, malt extract 10 g/L, dextrose 4 g/L, and agar 20 g/L) (Lin et al., 2018). The potato cultivar *S. tuberosum* L. Favorita was selected as plant material. It was introduced from the Netherlands and widely planted in China.

### 2.2 Growth-promoting, biocontrol, and yield-increasing efficacy of Y6 under greenhouse conditions

The growth-promoting efficacy of strain Y6 on potatoes was determined through pot experiments using potato cultivar *S. tuberosum* L. Favorita under artificial climate chamber (PQX-450B-22HM) conditions with 16 h of light and 8 h of darkness at 22°C. Each pot contained 1 kg soil (organic matter 22.7 g/kg, total nitrogen 0.79 g/kg, alkaline hydrolysis nitrogen 118.4 mg/kg, available phosphorus 1.61 mg/kg, and available potassium 18.6 mg/kg). The potato tubers were cut into small pieces, each with at least one bud, air-dried in the shade, and planted in soil at 22°C. The planting depth was 3–4 cm. Each treatment consisted of 8 potato plants, for a total of 16 plants, (a) Y6; (b) Control: no Y6; After potato seedlings emerged, the Y6-treated group was inoculated with 100 mL (1 × 10^7^ CFU/mL) of Y6 cells by root irrigation. The control group was treated with water 100 mL water. Potato plants were watered twice weekly with 50 mL water, and fertilizer (15-8-22, Ba tian China) was applied weekly after potato seedlings emerged. The plant height and stem thickness of the potato were measured after potato seedlings emerged 30 days.

The biocontrol efficacy of *B. velenzensis* Y6 against *S. scabies* was determined through pot experiments using potato cultivar *S. tuberosum* L. Favorita under greenhouse conditions. Each pot contained 10 kg soil (organic matter 22.7 g/kg, total nitrogen 0.79 g/kg, alkaline hydrolysis nitrogen 118.4 mg/kg, available phosphorus 1.61 mg/kg, and available potassium 18.6 mg/kg). Potato tuber pieces were planted in soil at 22°C, and the planting depth was 5–6 cm. After potato seedlings emerged for 30 days, the 100 mL (1 × 10^7^ CFU/mL) Streptomyces scabies was inoculated by root irrigation. For Y6 treatment, strain Y6 was cultivated for no more than 9 h on LB liquid medium at 37°C and 180 rpm/min, transferred to fresh LB liquid medium, and shaken to OD_600_ = 1 at 37°C, then rinsed twice with sterile water. Hundred mL containing 1 × 10^8^ Y6 cells were applied to the treatments. Each of the three treatments included four potato plants, for a total of 12 plants: (a) Y6 and *S. scabies* inoculated on the same day; (b) control: *S. scabies* only; (c) control-0: without Y6 or *S. scabies*; potato plants were watered twice weekly with 500 mL water, and fertilizer (15-8-22, Ba tian China) was applied weekly after potato seedlings emerged. Potato tubers were harvested 90 days after potato seedlings emerged, and the disease index for each treatment was calculated as originally described by Wanner et al. (2014) and Lin et al. (2018): ∑ (disease coverage x major disease type x number of tubers with these scores)/(18 × total number of potato tubers counted) × 100. Disease types were classified into four classes: 0 = no symptoms, 1 = superficial, 2 = raised, and 3 = depressed. Lesion coverage on each tuber was divided into seven classes: 0 = no scab, 1 = 0.1% to 2%, 2 = 2.1% to 5%, 3 = 5.1% to 10%, 4 = 10.1% to 25%, 5 = 25.1% to 50%, and 6 = 50%. Potato tubers with coverage of class 3 or higher were counted as diseased and the incidence of potato scab was counted. Potato yield was also measured for each treatment.

### 2.3 Growth-promoting, biocontrol, and yield-increasing efficacy of Y6 under field conditions

The field experiment was conducted on November 16, 2021, in Huidong County, Huizhou City, Guangdong Province (114° 72′ E, 22° 98′ 48″ N, 41 m a.s.l.), a site with annual rain totals of 1,600–2,300 mm and a mean annual air temperature of 22.6°C. Field trials were also conducted on December 4, 2021, in Dapeng New District, Shenzhen, China (114°50′ E, 22°59′ N, 61 m a.s.l.), a site with annual rain totals of 1846 mm and a mean annual air temperature of 22.5°C, and on January 7, 2023, at Zhucun, Zengcheng District, Guangzhou City, Guangdong Province (113°71′E, 23°98′ N, 3.6 m a.s.l.), where the annual rainfall was 2039.5 mm and the mean annual temperature was 22.1°C. The test plots had blocks 1.2 m wide (including the furrows), blocks 25–30 cm high, 20 cm between plants, 30 cm between rows within the blocks, and 5–6 cm sowing depth. There are three blocks per plot and three plots per treatment. After turning the ground and starting the ridge, the organic and compound fertilizer was applied in the middle of the furrow. Commercial organic fertilizer: 400 kg/mu; potato compound fertilizer (15–8-22): 100 kg/mu (Ba tian China). After cutting the seed potatoes, tuber pieces with a bud are mixed directly with the biofertilizer made of Y6. The specifications of the biofertilizer are: the number of active bacteria is more than 20 billion/g, and the dosage is 1 kg per mu.

To evaluate the effect of strain Y6 on the growth of potato roots, potato roots were randomly collected from the CK and Y6 treatments in the field at 60 days after potato seedlings emerged, and the roots were washed with Sterile water. Then the Sample roots were photographed with a Nikon camera (NIKON D300S), and the sample roots were scanned using a scanner (EPSON, the United States) with projected light, and the scanned images were analyzed with the winRHIZO root image analysis system (Regent instruments Inc., Canada) to obtain root morphological data such as total root length and root surface area.

Moreover, we collected 40 potato tubers randomly from the middle block of each plot to assess disease severity and incidence in each plot, and three plots of the same treatment were used to determine the mean ± standard error and compared with the other treatments through the Tukey test.

In addition, potato tubers were harvested 90 days after potato seedlings emerged to evaluate their yield. Potato tubers are classified according to weight: commercial potatoes >75 g, first-grade potatoes >175 g, second-grade potatoes 75 to 175 g, and non-commercial potatoes <75 g.

### 2.4 Extraction of lipopeptide from the clearance zone

**Figure 1:**
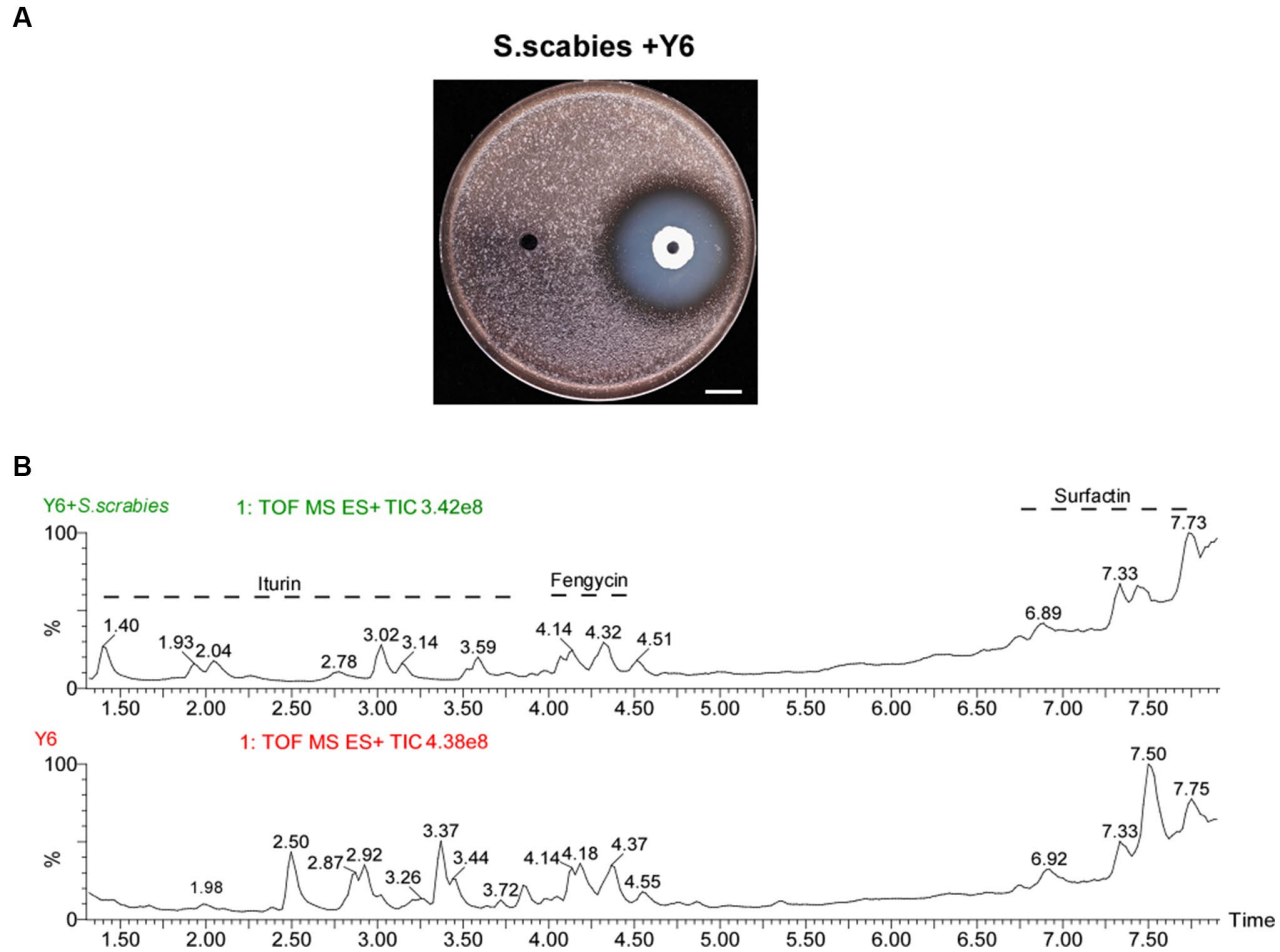
Antagonistic activity of Y6 against *S. scabies* and the analysis of lipopeptide crude extracts from Y6 by UPLC/Q-TOF. **(A)** Antagonistic activity of Y6 against *S. scabies* via a spot-on lawn assay. The white line indicated the size of the inhibition circle, measured after 24 h of incubation at 30°C (Y6, 32.0 ± 0.5 mm, mean ± SD, *n* = 3). The bar represents 10 mm. **(B)** Representative chromatograms of the LPs (iturin, fengycin, and surfactin) from strain Y6 using UPLC/Q-TOF analysis. After 24 h of incubation at 30°C, a 600 mg agar sample was collected from the clear area, which was minced and mixed with 2 mL of acetonitrile/water (1:1 v/v), shaken well, and sonicated twice for 30 s, then centrifuged. The supernatant was collected, filtered, and analyzed using UPLC/Q-TOF. As a control, LP was isolated in a similar way but without the addition of *S. scabies* in 0.7% YME agar.

We extract LPs from the clearance zone (Figure 1A) 0.200 μL of 10^7^ CFU/mL *S. scabies* cells were added to 4 mL of 0.7% YME agar (cooled to about 50°C) and mixed thoroughly, then poured onto YME plates (1.5% agar). After the agar solidified on the surface, two small holes (5 mm in diameter) were punched on each plate, and 40 μL of Y6 strain (OD_600_ = 0.4) grown in LB medium was added to each hole, and the liquid in the small holes was blown to complete dryness on an ultra-clean bench. After 24 h of incubation at 30°C and the appearance of a clear ring of inhibition, a 600 mg agar sample was collected from the clear area, which was minced and mixed with 2 mL of acetonitrile/water (1:1 v/v), shaken well, and sonicated twice for 30 s, then centrifuged, and the supernatant was collected and filtered. As a control, LP was isolated in a similar way but without the addition of *S. scabies* in 0.7% YME agar. Agar samples (600 mg) were collected around the holes (5 mm) (Cao et al., 2018).

### 2.5 Identification of LPs by UPLC/Q-TOF

The acetonitrile/water extracts were analyzed by reverse phase ultra-performance liquid chromatography coupled with time-of-flight mass spectrometry (UPLC/Q-TOF). The mass-to-charge ratio (*m*/*z*) was used to identify the lipopeptide compounds. A gradient elution with (A) acetonitrile (containing 0.1% formic acid) and (B) water (containing 0.1% formic acid) was utilized, with the column temperature being kept at 40°C. Following is how the gradient program was applied: 0 min, 40% A; 4.5 min, 80% A; 5.0 min, 80%A; 7 min,95%A;7.5 min, 98%A; 8 min, 40%A; and 10 min, 40% A. The flow rate of 0.3 mL/min was used. With a capillary voltage of 3.26 kV and a positive ionization mode selected, the Electrospray Ionization (ESI) source was kept at 150°C. Six hundred L/h of nitrogen was flowing. The flow of argon was 50 L/h (Cao et al., 2018).

### 2.6 Antibacterial activity of Y6 and its derived mutants against *Streptomyces scabies*

A spot-on-lawn experiment was used to assess the antibacterial activity of strain Y6 and its mutations against *Streptomyces scabies* (*S. scabies*). *S. scabies* was cultivated on a solid YME medium at 28°C for 14 days. Spores were collected throughout the plate with a sterile cotton swab and dissolved in 10 mL of sterile water, and spore concentration was determined by the gradient dilution method. Two hundred μL of 10^7^ CFU/mL *S. scabies* cells were added to 4 mL of 0.7% YME agar (cooled to about 50°C) and mixed thoroughly, then poured onto YME plates (1.5% agar). After the agar solidified on the surface, a small hole (5 mm in diameter) was punched on each plate, and 40 μL of Y6 strain (OD_600_ = 0.4) grown in LB medium was added to each hole, and the liquid in the small holes was blown to complete dryness on an ultra-clean bench. The size of the circle of inhibition after 24 h of incubation at 30°C was used to quantify antagonistic activity. The experiments were carried out at least three times (Cao et al., 2018).

### 2.7 Total RNA extraction and transcriptomic analysis

To evaluate the effect of Y6 on the transcriptome of potato, the following experiments were performed. Firstly, 8 samples were collected from the tender portion of the potato root system in each of the CK and Y6 treatments in the field at 60 days after potato seedlings emerged in Huidong County, Huizhou City, Guangdong Province (114° 72’ N, 22° 98′ 48″ E, 41 m a.s.l.). Roots were washed twice with sterile water and then rapidly frozen in liquid nitrogen, and the samples were finally stored in an ultra-low temperature refrigerator at −80°C. Then, total RNA was extracted using the Trizol Reagent (Invitrogen Life Technologies). The concentration, purity, and integrity of the RNA were then assessed using a NanoDrop spectrophotometer (Thermo Scientific). The RNA sample preparations started with three micrograms of RNA as input. Following the methods below, sequencing libraries were created. Using poly-T oligo-attached magnetic beads, mRNA was first separated from total RNA. Utilizing divalent cations at a high temperature in an exclusive fragmentation buffer made by Illumina, fragmentation was conducted. Super Script II and random oligonucleotides were used to create first-strand cDNA. Then, DNA Polymerase I and RNase H were used to create a second strand of cDNA. Exonuclease/polymerase activities were used to blunt the ends of any remaining overhangs after the enzymes were removed. Illumina PE adapter oligonucleotides were ligated to prepare for hybridization after the 3′ ends of the DNA fragments were adenylated. The library fragments were purified using Beckman Coulter’s AMPure XP Technology (Beverly, CA, United States) to choose cDNA fragments that were the desirable 400–500 bp in length. Using the Illumina PCR Primer Cocktail in a 15-cycle PCR reaction, DNA fragments with ligated adaptor molecules on both ends were specifically enriched. The Agilent high-sensitivity DNA assay was used on an Agilent Bioanalyzer 2100 system to quantify the products after they had been purified (AMPure XP system). On the Illumina NovaSeq 6000 platform, the sequencing library was subsequently sequenced.

Samples are sequenced on the platform to produce picture files, which are then processed by the platform’s software to produce the original data in FASTQ format (Raw Data). We filter the sequencing data using the Cutadapt (v1.15) software to obtain high-quality sequences (Clean Data) for further analysis because the sequencing data contains a lot of connections and low-quality Reads. SolTub_3.0 was used as the reference genome to create the genome of Solanum_tuberosum. Every gene was assigned a term in the Gene Ontology database, and the number of differentially enriched genes was calculated for each term. Calculate the *p*-value using the hypergeometric distribution approach while using topGO (2.40.0) to perform GO enrichment analysis on the differential genes (the benchmark for substantial enrichment is *p*-value 0.05). Additionally, locate the GO word that is markedly enriched by differential genes to ascertain the primary biological functions carried out by differential genes. The enrichment analysis of the KEGG pathway of differentially expressed genes was performed using ClusterProfiler (3.16.1) software, with a particular emphasis on the significant enrichment pathway with *p*-value.

### 2.8 Statistical analysis

Statistical analyses in the experiments were performed using the t-test in IBM SPSS Statistics 20, with P 0.05 indicating significant differences. GraphPad Prism 8 was used to create the columns.

## 3 Results

### 3.1 *Bacillus velezensis* strain Y6 showed strong antibacterial activity against *Streptomyces scabies*

To investigate the antibacterial activity of *B. velezensis* strain Y6 against *S. scabies*, a spot-on-lawn assay was carried out with strain Y6. The antagonistic assay result in Figure 1A revealed a noticeable clearing zone of 32.0 ± 0.5 mm in diameter against *S. scabies*, indicating that strain Y6 has excellent antibacterial activity.

### 3.2 Identification of Y6-secreted lipopeptide compounds

**Figure 2:**
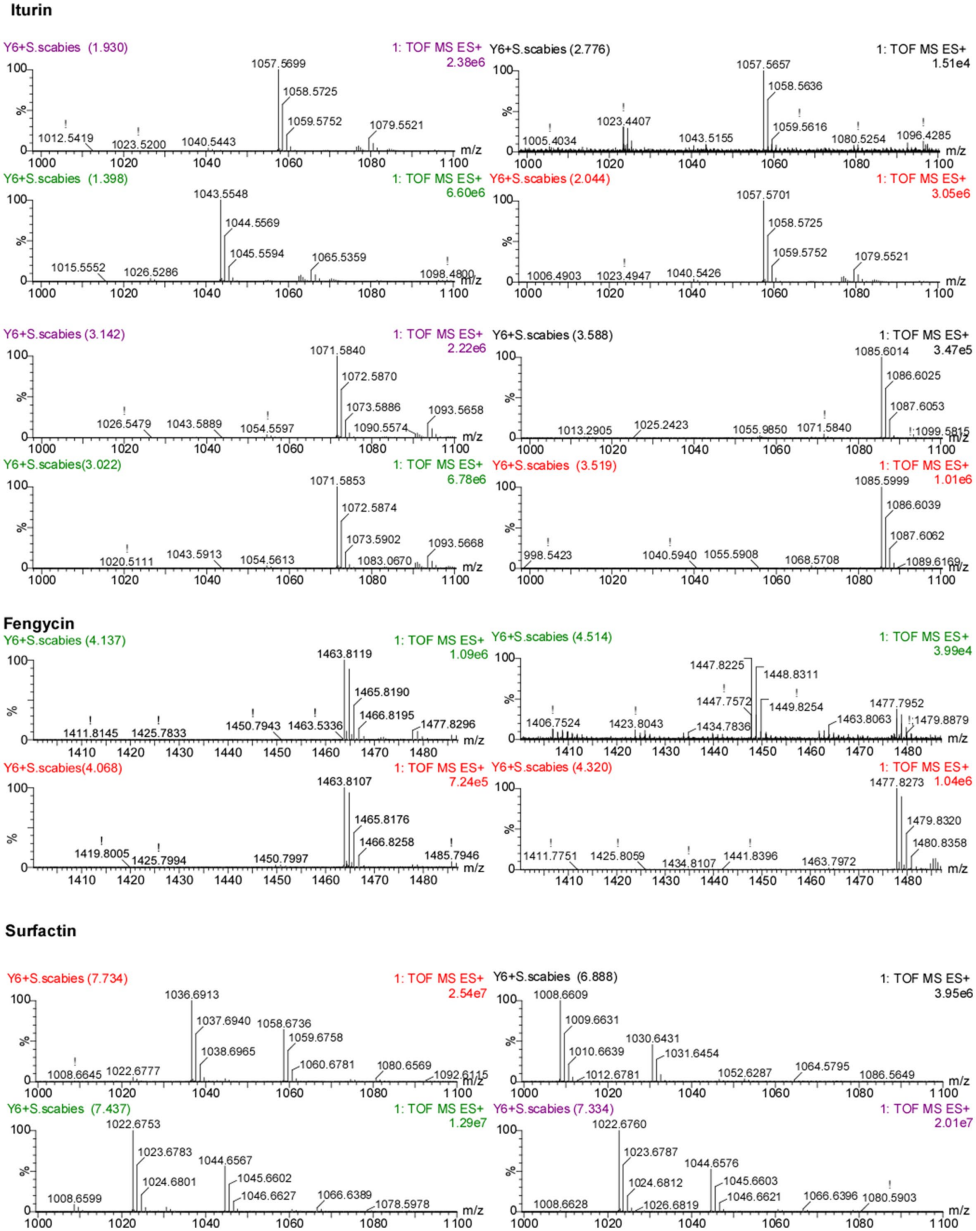
Identification of LP compounds in Y6 extracts using UPLC/Q-TOF analysis. The figure shows the mass spectra [M + H]^+^ for LPs, iturin, fengycin, and surfactin.

The antimicrobial activity of *B. velezensis* is related to its production of lipopeptide (LP) compounds. Using an acetonitrile/water-based method we obtained LP crude extracts from transparent inhibitory rings (Figure 1A), which were then subjected to UPLC/Q-TOF analysis. The mass spectrometry results showed three classes of compounds: iturin, fengycin, and surfactin (Figure 1B). Depending on the length of the terminal fatty acid carbon chain, multiple isomers existed for each LP, among which we found eight isomers (C14 to C17) for iturin, four isomers (C14 to C15) for fengycin, and four isomers (C13 to C15) for surfactin according to the retention time of the peaks of the chromatograms (Figure 2).

Meanwhile, we also found that *S. scabies* was able to cause changes in the lipopeptides secreted by Y6, and the results showed that in the presence of the pathogen, iturin was more altered, with the appearance of new isomers, and fengycin was not altered much whereas the relative amount of C15 isoform in surfactin was increased in comparison to the control (Figure 1B).

### 3.3 Antibacterial activity of Y6 and its derived mutants against *Streptomyces scabies*

**Figure 3:**
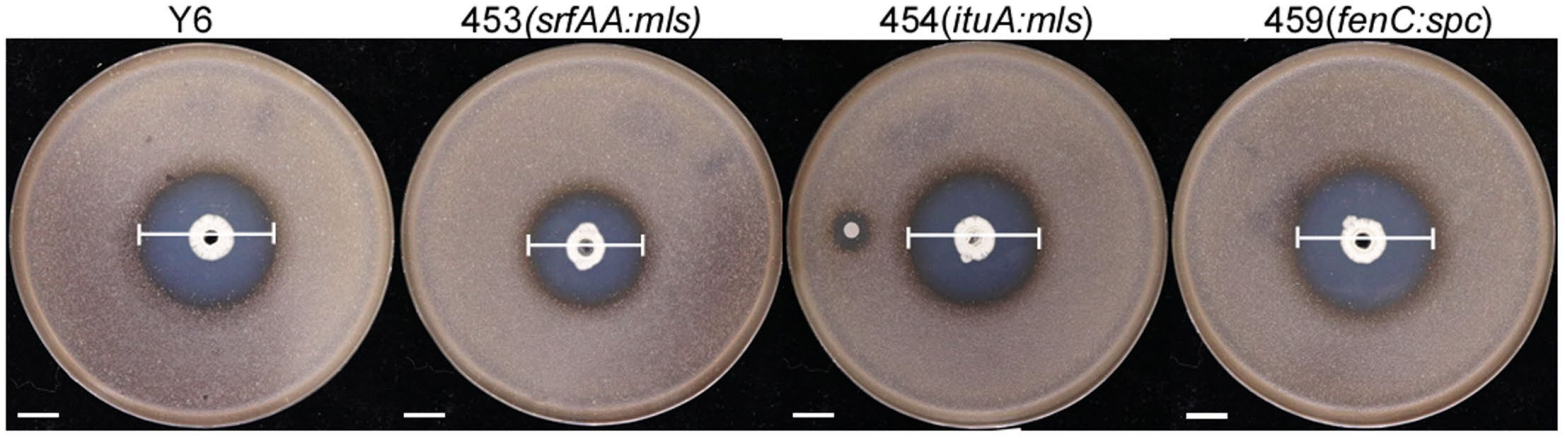
The antagonistic activity of the strain Y6 and mutants (453 (*srfAA:mls*), 454 (*ituA:mls*), 459 (*fenC:spc*)). The white line indicated the size of the inhibition circle, measured after 24 h of incubation at 30°C (Y6: 32.50 ± 0.50 mm, 453: 27.05 ± 0.05 mm, 454: 30.00 ± 0.50 mm, 459: 32.75 ± 0.43 mm, mean ± SD with *n* = 3). The bar represents 10 mm. The clearance zone indicated by the white lines was measured after 24 h incubation at 30°C.

To find out which lipopeptide played the main role in the Y6 antagonism against *S. scabies*, a spot-on-lawn assay was carried out with the strain Y6 and three mutants (453 (*srfAA:mls*), 454 (*ituA:mls*), and 459 (*fenC:spc*)). From Figure 3, it could be found that strain Y6 had very strong antagonistic activity against *S. scabies* with an inhibition circle of 32.5 ± 0.5 mm in diameter. Compared to the strain Y6, the mutant 453 (*srfAA:mls*) had a 16.92% reduction in the diameter of the clearance zone, the mutant 454 (*ituA:mls*) had a 6.15% reduction in the diameter of the clearance zone, and the diameter of the clearance zone of mutant 459 (*fenC:spc*) was nearly unchanged. These results indicated that the lipopeptides surfactin and iturin were required for Y6 antagonism against *S. scabies*.

### 3.4 Biocontrol and yield-increasing efficacy of Y6 under greenhouse conditions

**Figure 4:**
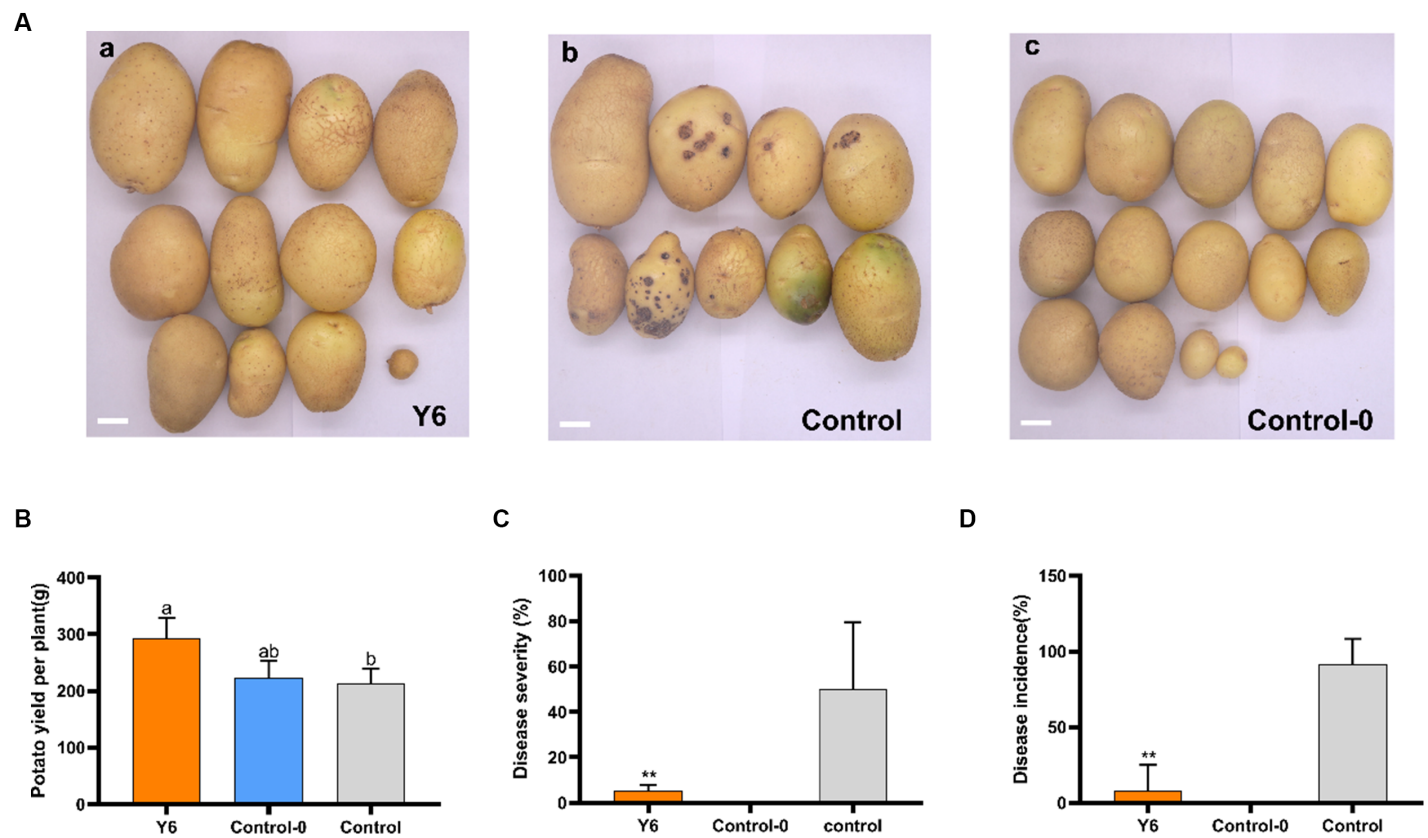
Biocontrol and yield-increasing efficacy of Y6 under greenhouse conditions. **(A)** Potato tubers harvested from three treatments: **(a)** inoculated with Y6 and *S. scabies* on the same day; **(b)** inoculated with *S. scabies* only; **(c)** control without Y6 or *S. scabies*. The bar represents 2 cm. **(B)** Potato yields from three treatments. **(C)** Disease severity of potato common scab. **(D)** Disease incidence of the potato common scab. *p*-values were calculated using Tukey’s test. Asterisks (**) indicate *p* < 0.01 as compared to the treatment of control.

To determine if Y6 could lessen the severity of common potato scab and increase yield, pot experiments were conducted. Twelve potato plants were divided into 3 treatments: (a) inoculated with Y6 and *S. scabies* on the same day; (b) inoculated with *S. scabies* only; and (c) inoculated without Y6 or *S. scabies*. The disease severity was determined by multiplying the percentage disease coverage index by the index of major disease types divided by 18, and the disease incidence was determined by dividing the number of diseased potatoes by the total number of potatoes. Potato tubers were harvested 90 days after potato seedlings emerged. Potato yields were also counted. It could be found from Figures 4A,C that treatment with Y6 could significantly reduce the disease severity of potato common scab from 49.92 ± 25.74% (inoculated with *S. scabies* only) to 5.56 ± 1.89% (inoculated with *S. scabies* and Y6 on the same day) (*p* < 0.01, Tukey’s test). The disease incidence of potato common scab could be reduced from 91.67 ± 14.42% (inoculated with *S. scabies* only) to 8.33 ± 14.43% after treating with Y6 (Figure 4D). Meanwhile, the treatment with Y6 could also increase the potato yield by 37.32% compared with the control (inoculated with *S. scabies* only) (Figure 4B). The above results indicated that Y6 could lessen the disease severity of potato common scab and increase potato yields in pot trials.

### 3.5 Biocontrol and yield-increasing efficacy of Y6 under field conditions

**Figure 5:**
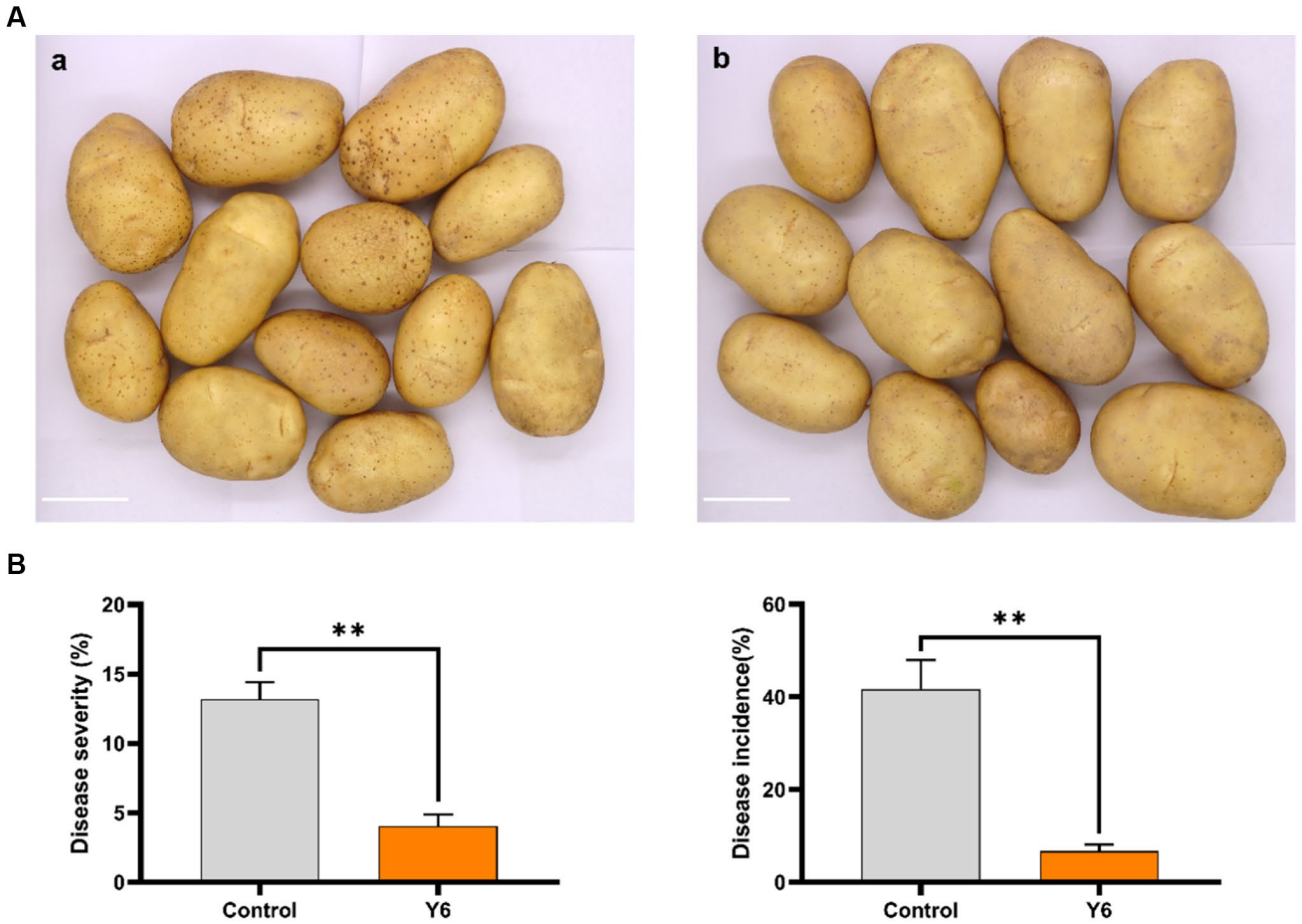
Biocontrol and yield-increasing efficacy of Y6 under field conditions. **(A)** Potato tubers were harvested from two treatments: **(a)** inoculated with Y6, tuber pieces with a bud were mixed with the biofertilizer made of Y6 before planting in soil; **(b)** no treatment. The bar represents 5 cm. **(B)** Data about the disease severity and disease incidence of the potato common scab from two treatments. One hundred twenty randomly chosen tubers from each treatment were used to calculate the severity of the disease as a percentage. We collected 40 potato tubers randomly from the middle block of each plot to assess disease severity and incidence in each plot, and three plots of the same treatment were used to determine the mean ± standard error and compare with the other treatments. *p*-values were calculated using Tukey’s test. Asterisks (**) indicate *p* < 0.01 as compared to the treatment of control.

To evaluate the effect of strain Y6 on potato scab and yield in a natural environment, a field trial was carried out in fields with potato common scab that occurs naturally. Due to the rice and potato crop rotation pattern used in this field, we observed a low disease severity of common scab in harvested potatoes. However, compared to the control, Y6 treatment significantly reduced disease severity from 13.20 ± 1.00% to 4.00 ± 0.70% (*p* < 0.01; Tukey’s test) and decreased disease incidence from 41.70 ± 5.10% to 6.70 ± 1.20% (Figure 5). Moreover, it could be found that strain Y6 could promote the yield of potato. At the trial site in Huidong, Huizhou, compared with the control group, the yield of potato in the Y6-treated group increased by 14.05%, among them, the yield of commercial potato in the Y6-treated group significantly increased by 23.40% (*p* < 0.05). The yield of first-grade potato in the Y6-treated group increased by 41.38% (Table 1). At the trial site in Dapeng, Shenzhen, the yield of commercial and first-grade potatoes increased significantly after Y6 inoculation compared to the control (*p* < 0.05). The yield of commercial potato in the Y6-treated group significantly increased by 17.29%, and the yield of first-grade potato in the group treated with strain Y6 increased by 40.99% (Table 1). At the trial site in Zengcheng, Guangzhou, compared with the control group, the yield of potato in the Y6-treated group significantly increased by 39.06% (*p* < 0.01). Among them, the yield of commercial potato in the Y6-treated group significantly increased by 40.75% (*p* < 0.01), and the yield of secondary potato significantly increased by 70.63% (*p* < 0.01) (Table 1). The above results indicated that Y6 could be used for biological control of common scab of potato and increase potato yields in field trials.

**Table 1:** Effects of strain Y6 on the yield of potato in different fields.

Filed		Control (t/mu)	Y6 (t/mu)	Yield increase rate (%)
Huidong	Potato yield	1.21 ± 0.21a	1.38 ± 0.19a	14.05%
	Commercial potato yield	0.94 ± 0.25b	1.16 ± 0.21a	23.40%
	First grade potato yield	0.29 ± 0.17a	0.41 ± 0.17a	41.38%
	Secondary potato yield	0.67 ± 0.08a	0.72 ± 0.07a	7.46%
	Non-commercial potato yield	0.25 ± 0.07a	0.23 ± 0.06a	−8.00%
Dapeng	Potato yield	2.96 ± 0.43a	3.25 ± 0.63a	9.78%
	Commercial potato yield	2.66 ± 0.30b	3.12 ± 0.38a	17.29%
	First grade potato yield	1.61 ± 0.42b	2.27 ± 0.71a	40.99%
	Secondary potato yield	1.07 ± 0.18 a	0.84 ± 0.53 a	−21.50%
	Non-commercial potato yield	0.28 ± 0.11a	0.14 ± 0.10b	−50.00%
Zengcheng	Potato yield	2.07 ± 0.10b	2.87 ± 0.28a	39.06%
	Commercial potato yield	1.83 ± 0.11b	2.57 ± 0.27a	40.75%
	First grade potato yield	1.03 ± 0.10a	1.21 ± 0.14a	17.85%
	Secondary potato yield	0.80 ± 0.11b	1.37 ± 0.19a	70.63%
	Non-commercial potato yield	0.24 ± 0.08a	0.30 ± 0.11a	25.52%

Commercial potatoes >75 g, first-grade potatoes >175 g, second-grade potatoes 75 to 175 g, and non-commercial potatoes <75 g. Means followed by various letter (s) within the same column are significantly different from one another (*p* ≤ 0.05).

### 3.6 Transcriptome profiling effects of strain Y6 on potato

**Figure 6:**
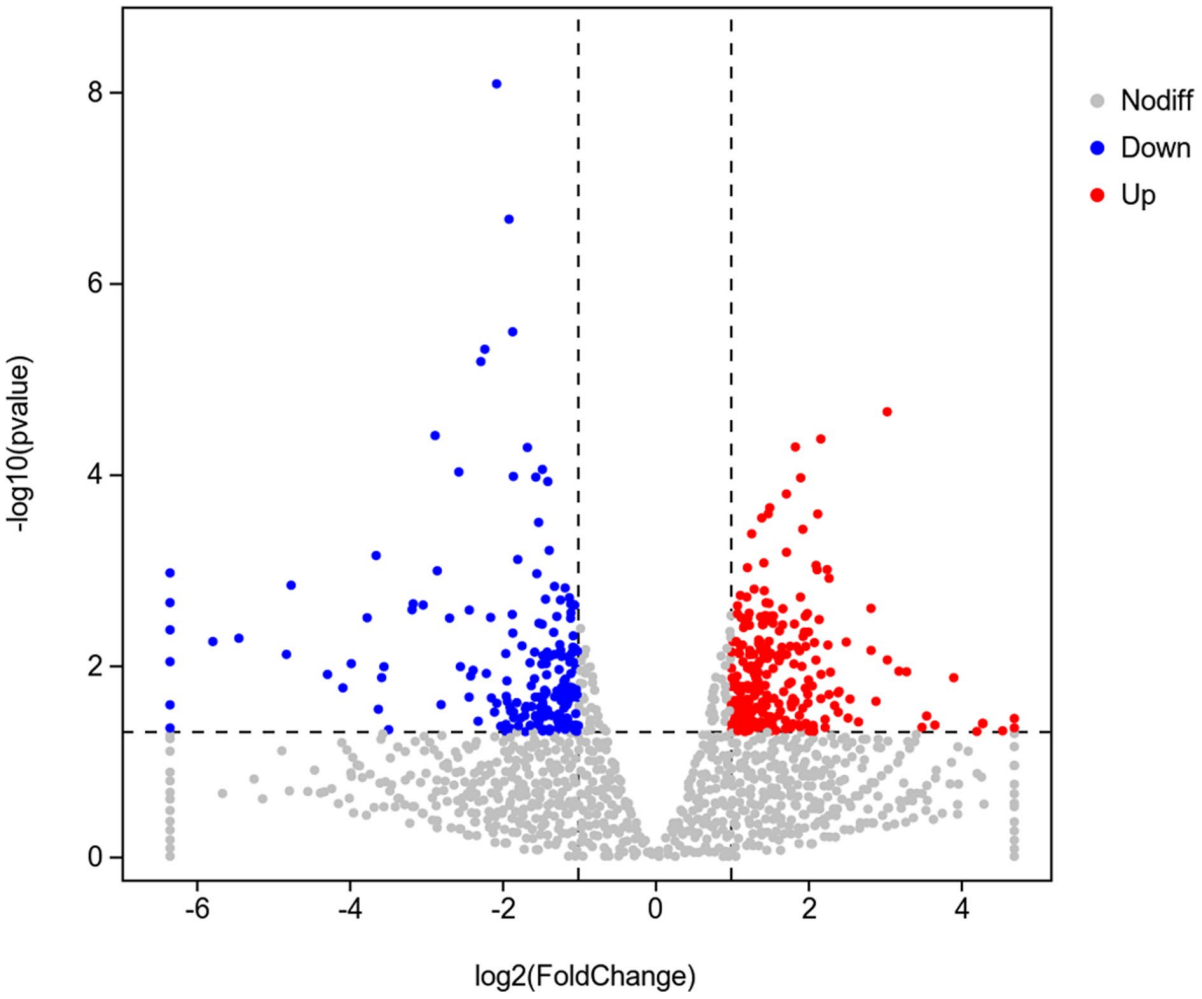
Volcano diagram of differentially expressed genes (DEGs) in the transcriptome of plants inoculated with strain Y6 and control. Red dots indicate gene up-regulation and blue dots indicate gene down-regulation.

**Figure 7:**
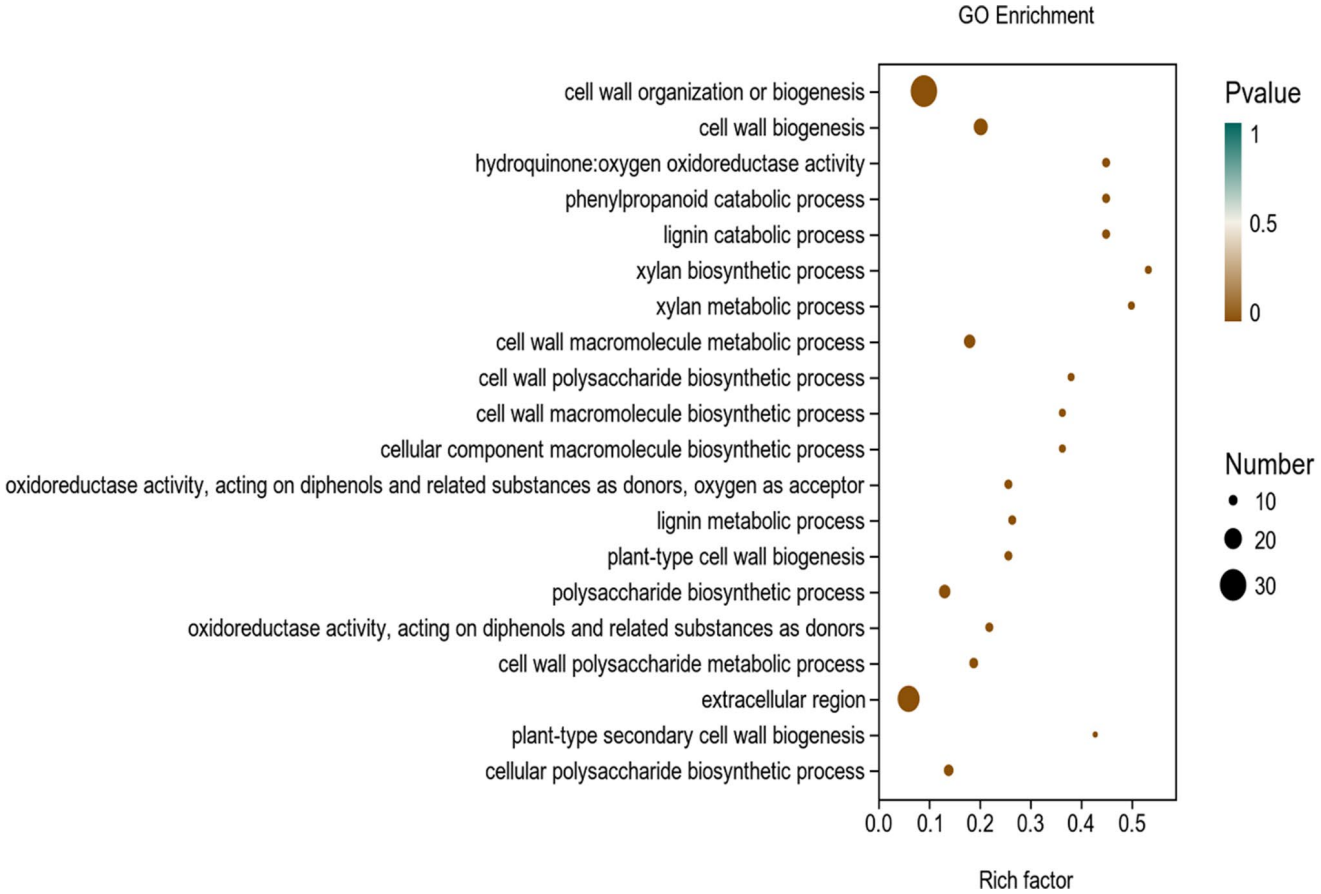
GO enrichment analysis of differentially expressed genes (DEGs) in the transcriptome of plants inoculated with strain Y6 and control. The *X*-axis represents the enrichment factor. The *Y*-axis represents the GO term name.

To investigate the mechanism of Y6 on the biocontrol and growth promotion of potato, we analyzed the transcriptomes of potato roots after inoculating the strain Y6 first. The result showed that there were 256 genes upregulated and 183 genes down-regulated in potato roots compared with those in uninoculated roots (Figure 6). Moreover, we also performed enrichment analysis of the DEGs in potato using the GO database (Figure 7). A total of 8,105 genes were enriched in 987 categories of three major categories in the GO database. Among the DEGs of potato, most of the enriched metabolic processes are related to the cell wall. Thirty genes were enriched in the cell wall organization or biogenesis. About 8 genes were enriched in the xylan biosynthetic or xylan metabolic process. Up to 25 genes were enriched in the extracellular region. There were 9 genes enriched in hydroquinone oxygen oxidoreductase activity.

**Figure 8:**
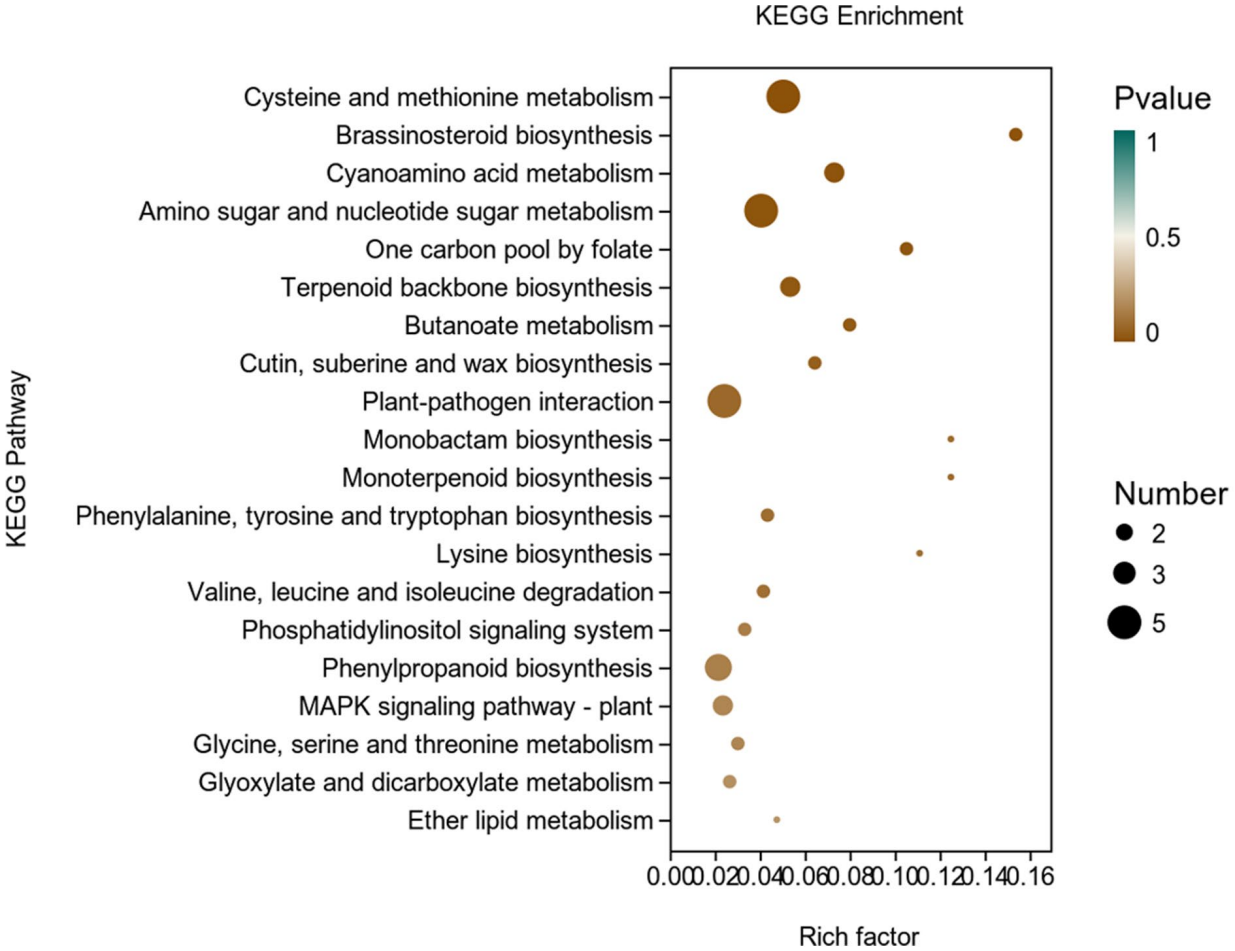
KEGG enrichment analysis of differentially expressed genes (DEGs) in the transcriptome of plants inoculated with strain Y6 and control. The *X*-axis represents the enrichment factor. The *Y*-axis represents the pathway name.

In addition, we used the KEGG database to carry out an enrichment analysis of DEGs in potato (Figure 8).There were 77 genes classified into 45 KEGG functional pathway categories. Overall, the maximum annotated genes were found to be associated with KEGG pathways like cysteine and methionine metabolism; brassinosteroid biosynthesis; cyanoamino acid metabolism; amino sugar and nucleotide sugar metabolism; one carbon pool by folate; terpenoid backbone biosynthesis; plant-pathogen interaction; phosphatidylinositol signaling system; MAPK signaling pathway, etc.

#### 3.6.1 Strain Y6 promoted the growth of potato

Potato pot experiments and field trials were conducted to verify the growth-promoting function of strain Y6. We found that the height of potatoes inoculated with strain Y6 increased by 12.08% at 30 days compared to the control (Supplementary Figures S1A,B) under artificial climate chamber conditions. In the field trials, inoculation with strain Y6 increased the germination rate of potato tubers from 90.61 ± 7.76% to 94.38 ± 2.11% but had no effect on the height of the potato plants at 30 days (Supplementary Figure S2). We found that the yield of potatoes per plant increased by 11.11% and the fresh weight of potato plants increased by 33.49% after inoculation with strain Y6 compared to the control at 60 days (Supplementary Figure S3). We also found that Y6 could promote the growth of potato roots, especially lateral roots were promoted after inoculation with strain Y6 (Supplementary Figure S1C). Compared with the control group, the root length in the Y6-treated group was increased by 42.16% at 60 days (Supplementary Figure S1D). The effects of strain Y6 on potato root surface area were significant (*p* < 0.05) at 60 days after inoculation; in the Y6-treated group, the potato root surface area was 74.93% bigger than the control (Supplementary Figure S1E).

##### 3.6.1.1 Strain Y6 induces the expression of potato growth-related genes

Brassinosteroid (BR) hormones are indispensable for root growth, and BR biosynthesis is largely restricted to the root elongation zone (Vukasinovic et al., 2021). The result of transcriptome profiling showed that after inoculating with strain Y6, BR biosynthesis genes in potato were up-regulated; the gene (PGSC0003DMG400017354) encoding cytochrome P450 family protein and the gene (PGSC0003DMG400005693) encoding castasterone 26-hydroxylase were up-regulated 1.22 and 1.47 fold, respectively, compared with those in potato roots without inoculation of strain Y6. Moreover, there were many genes involved in cell wall metabolism. Interaction with strain Y6 increased the expression level of cell wall organization or biogenesis genes in potato roots 1.01–3.49 fold. The expression of lignin catabolic process-related genes was up-regulated 1.13–2.15 fold. The expression of xylan biosynthetic process-related genes was up-regulated 1.01–2.13 fold. The cell wall macromolecule metabolic process-related genes were also up-regulated 1.24–1.58 fold after being cultured with strain Y6 in potato roots (Table 2).

**Table 2:** Effects of strain Y6 on the genes related to cell wall metabolism in potato.

GO ID	Term	Gene ID	Log2 fold change	*p*-value	Gene description
GO:0071554	Cell wall organization or biogenesis	PGSC0003DMG400001031	1.35	0.29 × 10^−1^	Protein COBRA
		PGSC0003DMG400001769	1.72	0.08 × 10^−1^	Beta-1,3-glucuronyltransferase
		PGSC0003DMG400003822	1.35	0.01 × 10^−1^	Cellulose synthase
		PGSC0003DMG400006497	1.12	0.02 × 10^−1^	Conserved gene of unknown function
		PGSC0003DMG400006868	1.90	0.02 × 10^−1^	MYB20
		PGSC0003DMG400008016	1.44	0.16 × 10^−1^	Glycogenin
		PGSC0003DMG400009381	1.80	0.22 × 10^−1^	NAC domain protein
		PGSC0003DMG400010075	1.96	0.41 × 10^−1^	Zinc finger protein
		PGSC0003DMG400011148	1.42	0.08 × 10^−1^	Cellulose synthase 3
		PGSC0003DMG400014401	1.29	0.08 × 10^−1^	Glycosyltransferase, CAZy family GT8
		PGSC0003DMG400016038	1.15	0.09 × 10^−1^	Polygalacturonase
		PGSC0003DMG400016302	1.40	0.00 × 10^−1^	Class IV chitinase
		PGSC0003DMG400016880	1.36	0.06 × 10^−1^	Gene of unknown function
		PGSC0003DMG400019228	1.52	0.03 × 10^−1^	Glycosyltransferase, CAZy family GT8
		PGSC0003DMG400020374	2.08	0.49 × 10^−1^	Polygalacturonase
		PGSC0003DMG400021602	1.24	0.23 × 10^−1^	Chitinase
GO:0071554	Cell wall organization or biogenesis	PGSC0003DMG400024530	1.98	0.41 × 10^−1^	Protein COBRA
		PGSC0003DMG400024989	1.45	0.08 × 10^−1^	Nucleic acid binding protein
		PGSC0003DMG400025474	1.35	0.17 × 10^−1^	Nucleic acid binding protein
		PGSC0003DMG400026189	1.58	0.48 × 10^−1^	Xyloglucan endotransglycosylase/hydrolase 16 protein
		PGSC0003DMG400028426	1.62	0.09 × 10^−1^	Cellulose synthase catalytic subunit
		PGSC0003DMG400028674	1.79	0.27 × 10^−1^	Pectinesterase-2
		PGSC0003DMG400030191	3.49	0.45 × 10^−1^	Pectinesterase
		PGSC0003DMG40003Y63	1.01	0.14 × 10^−1^	Conserved gene of unknown function
		PGSC0003DMG400032772	1.30	0.12 × 10^−1^	Protein COBRA
		PGSC0003DMG402024882	2.13	0.00 × 10^−1^	Conserved gene of unknown function

Genes for early plant response to auxin are usually divided into three categories: Aux/IAAs, GH3, and SAURs (small auxin-up RNAs) (Liu et al., 2005, 2020). After inoculating with strain Y6, two genes (PGSC0003DMG400005236, PGSC0003DMG400001667) encoding SAUR family proteins in potato were up-regulated 1.97 and 4.29 fold, respectively.

##### 3.6.1.2 Strain Y6 affects the expression of genes involved in potato metabolism

Differential expression of some metabolism-related genes was caused after inoculation of potato plants with strain Y6. The photosynthetic pathway-related gene PGSC0003DMG400024016 was up-regulated by 3.19 times, and the Citrate cycle (TCA cycle) pathway-related gene PGSC0003DMG400024016 was down-regulated 1.57 fold. There were 12 DEGs related to amino acid metabolism. Four genes were up-regulated, while 8 genes were down-regulated. Genes (PGSC0003DMG400021476, PGSC0003DMG400004572, PGSC0003DMG400033126, and PGSC0003DMG400004660), were up-regulated 1.19–1.62 fold. And 8 genes were down-regulated 1.02–2.20 fold (Table 3). This metabolism-related differential expression may promote the accumulation of photosynthetic products and contribute to higher yields in potato.

**Table 3:** Effects of strain Y6 on the expression of genes involved metabolic pathways in potato.

	Gene ID	Log2 fold change	*p*-value	Gene description
Up-regulated	PGSC0003DMG400021476	1.62	0.25 × 10^−1^	1-aminocyclopropane-1-carboxylate oxidase 2
	PGSC0003DMG400004572	1.19	0.26 × 10^−1^	S-adenosyl-L-homocysteine hydrolase
	PGSC0003DMG400033126	1.26	0.28 × 10^−1^	Aspartate kinase
	PGSC0003DMG400004660	1.27	0.30 × 10^−1^	Serine hydroxymethyltransferase
Down-regulated	PGSC0003DMG400006506	−1.14	0.37 × 10^−1^	beta-cyanoalanine synthase 1
	PGSC0003DMG400012321	−1.02	0.49 × 10^−1^	1-amino-cyclopropane-1-carboxylate synthase
	PGSC0003DMG400020334	−2.20	0.12 × 10^−1^	Prephenate dehydrogenase
	PGSC0003DMG400023957	−1.52	0.39 × 10^−1^	Prephenate dehydrogenase
	PGSC0003DMG400012522	−1.22	0.08 × 10^−1^	3-hydroxy-3-methylglutaryl coenzyme A synthase
	PGSC0003DMG401017380	−1.11	0.17 × 10^−1^	Cytosolic acetoacetyl-coenzyme A thiolase
	PGSC0003DMG400008000	−1.46	0.47 × 10^−1^	L-asparaginase
	PGSC0003DMG400026671	−1.28	0.03 × 10^−1^	Arginine decarboxylase

#### 3.6.2 Strain Y6 enhances systemic resistance in potato

##### 3.6.2.1 Strain Y6 induces the expression of transcription factors involved in plant stress tolerance

The *bHLH* transcription factor serves a critical function in the regulation of the growth and development of plants (Zhang et al., 2018). After inoculation with strain Y6, the expression of the bHLH transcription factor-related gene (PGSC0003DMG401022600) was up-regulated by 4.21 times in potato roots. Inoculation with strain Y6 also induced changes in the expression of other transcription factors associated with abiotic stress responses in potato roots, such as MYB and NAC transcription factors (Puranik et al., 2012). The MYB transcription factor genes (PGSC0003DMG400006868, PGSC0003DMG400004780, and PGSC0003DMG400007360) were up-regulated 1.21–1.90 fold compared with those in potato roots not cultured with strain Y6. The NAC transcription factor genes (PGSC0003DMG400009381, PGSC0003DMG400012113) were upregulated 1.80–1.89 fold.

##### 3.6.2.2 Strain Y6 increased the expression of genes related to secondary metabolism in potato

Many secondary metabolite products have the potential to improve plant responses to biotic stresses. It could be found that after inoculation with strain Y6, nine hydroquinone genes were up-regulated 1.13–2.15 fold (Table 4). The genes encoding diphenol oxidase, PGSC0003DMG400011019, PGSC0003DMG400019185, and PGSC0003DMG400020345 were up-regulated 1.63, 2.15, and 1.48 fold, respectively. The expression of genes encoding laccase (PGSC0003DMG400027168, PGSC0003DMG401027116, and PGSC0003DMG402027116) was up-regulated 1.13–2.11 fold in potato roots cultured with strain Y6 compared with those cultured without strain Y6. The up-regulation of hydroquinone genes contributed to enhancing the resilience of potato roots, increasing their resistance to pests and pathogens.

**Table 4:** Effects of strain Y6 on the expression of hydroquinone related genes in potato.

GO ID	Term	Gene ID	Log2 fold change	*p*-value	Gene description
GO:0052716	Hydroquinone: oxygen oxidoreductase activity	PGSC0003DMG400003512	1.24	0.04 × 10^−1^	Laccase
		PGSC0003DMG400011019	1.63	0.04 × 10^−1^	Diphenol oxidase
		PGSC0003DMG400019185	2.15	0.03 × 10^−1^	Diphenol oxidase
		PGSC0003DMG400020345	1.48	0.26 × 10^−3^	Diphenol oxidase
		PGSC0003DMG400020355	1.55	0.03 × 10^−1^	Conserved gene of unknown function
		PGSC0003DMG400027168	2.09	0.06 × 10^−1^	Laccase 90a
		PGSC0003DMG401008903	1.43	0.01 × 10^−1^	Conserved gene of unknown function
		PGSC0003DMG401027116	1.13	0.03 × 10^−1^	Laccase 90c
		PGSC0003DMG402027116	2.11	0.01 × 10^−1^	Laccase 90d

## 4 Discussion

In this study, we found that strain Y6 could reduce the severity of potato common scab by secreting lipopeptides and increasing systemic resistance in potato plants, and also increased potato yields. It was found that surfactin secreted by *Bacillus amyloliquefaciens* Ba01 was important for the control of *S. scabies* (Feng et al., 2022), and that the absence of surfactin and fengycin can cause the loss of antimicrobial activity in *Bacillus subtilis* YPS-32 (Zhou et al., 2022). In our study, the lipopeptides surfactin and iturin were required in Y6’s antagonism against *S. scabies*. Meanwhile, we found that *S. scabies* can induce Y6 to create new lipopeptide homologs (Figure 1B), and these new homologs may have stronger antimicrobial efficacy. The retention times of the peaks of the new substances in the chromatograms are different under the condition of unchanged molecular mass, which may be due to the new structural changes, and what kind of changes have happened to make lipopeptide antimicrobial activity improved needs to be further investigated.

The analysis of the transcriptome of potato roots showed that quite a lot of genes were differently expressed in response to strain Y6 inoculation. Genes encoding protein COBRA, cellulose synthase, MYB20, carbohydrate-active enzyme glycosyltransferase family 8, and xyloglucan endotransglucosylase/hydrolases (XTHs) were up-regulated to varying degrees by inoculating with strain Y6 (Table 2). These genes are all involved in the cell wall’s organization or biogenesis. Protein COBRA is an extracellular glycosyl-phosphatidyl inositol-anchored protein that controls anisotropic growth in Arabidopsis via its participation in cellulose microfibril orientation (Roudier et al., 2005). Cellulose synthase plays an important role in the synthesis of the primary cell wall, and the regulation of primary cell wall growth is essential for plant growth in response to environmental stress (Kesten et al., 2017). MYB transcription factors MYB20 is a transcriptional regulator that directly activates lignin biosynthesis genes and Phe biosynthesis genes during secondary wall formation in *Arabidopsis* (*Arabidopsis thaliana*) (Geng et al., 2020). The Carbohydrate-active enzyme glycosyltransferase family 8, known to be involved in plant cell wall biosynthesis，can promote the synthesis of pectin and hemicellulose (Yin et al., 2010). Xyloglucan endotransglucosylase/hydrolases (XTHs) are enzymes involved in the modification of load-bearing cell wall components and may contribute to the strengthening of the lateral wall of root hairs and the cell wall of the root differentiation zone after the completion of cell expansion (Maris et al., 2009). High expression levels of these genes could significantly promote the growth of potato. Moreover, brassinosteroid (BR) hormones are indispensable for root growth (Vukasinovic et al., 2021), after inoculating with strain Y6, the expression of BR-related genes in potato roots was up-regulated and the concentration of BR increased, which promoted the growth of potato roots.

The transcriptome analysis of potato inoculated with strain Y6 also revealed that strain Y6 enhances auxin signal transduction in potato. Small auxin up RNAs (SAURs) are a family of early auxin-responsive genes, SAURs involved in plant growth by affecting auxin synthesis and transport (Xu et al., 2017). In general, SAUR gene expression was upregulated by treatments or conditions that promoted growth and downregulated by factors that repressed growth (Du et al., 2020). The high expression level of SAUR genes promoted the growth of potato.

In the transcriptome of potato interacted with stain Y6, genes encoding beta-1,3-glucuronyltransferase, chitinases, pectinesterase, diphenol oxidase, and laccase were up-regulated in varying degrees. Beta-1,3-glucuronyltransferase, plays key roles in cell division, and trafficking of materials through plasmodesmata, in withstanding abiotic stresses (Balasubramanian et al., 2012). Plant chitinases play a key role in combating biotic and abiotic stresses. Plant chitinases were strongly expressed when plant (host) cells were under pathogen stress or, various abiotic stresses such as plant trauma, osmotic pressure, cold, heavy metal stress, and salt (Vaghela et al., 2022). The regulation of cell wall mechanical stability during fruit ripening, stem lengthening, tuber yield, and root formation is influenced by pectinesterase (Fries et al., 2007). It has also been demonstrated that pectinesterase contributes to the plant’s defense against pathogen attack. Laccase and diphenol oxidase play crucial roles in plant resistance to insects and fungi (Walker and Ferrar, 1998). High expression levels of these genes enhance the adaptive capacity of potato to environmental stresses.

## 5 Conclusion

In this study, we found that *B. velezensis* strain Y6 could reduce the severity of potato common scab, mainly through the secretion of lipopeptides (surfactin and iturin) and the enhancement of the plant’s resistance to environmental stresses, and also promoted potato growth by inducing a significant up-regulation of the expression of genes related to cell wall organization or biogenesis in the potato root system. Our study will help to reveal the molecular mechanism of biological control of potato common scab and growth promotion of potato by *B. velezensis* Y6 and provide a theoretical basis for the application of *B. velezensis* strain Y6 as a special PGPR for potato.

## Data availability statement

The data presented in the study are deposited in the NCBI repository, accession number PRJNA1025131.

## Author contributions

HT: Conceptualization, Methodology, Data curation, Software, Writing—original draft, Writing—review & editing. SW: Methodology, Data curation, Formal analysis, Writing—review & editing. XL: Methodology, Data curation, Formal analysis, Writing—review & editing. XL: Methodology, Data curation, Formal analysis, Writing—review & editing. JC: Methodology, Data curation, Formal analysis, Writing—review & editing. LZ: Methodology, Data curation, Formal analysis, Writing—review & editing. JW: Methodology, Data curation, Formal analysis, Writing—review & editing. JZ: Writing—review & editing, Project administration, Funding acquisition. YQ: Writing—review & editing, Project administration, Funding acquisition. XX: Writing—review & editing, Project administration, Funding acquisition. YC: Writing—review & editing, Project administration, Funding acquisition.

## Supplementary material

SUPPLEMENTARY FIGURE S1 Growth-promoting efficacy of Y6 on potato. **(A)** Representative photographs of potato plants to show the effects of the strain Y6 on the growth of potato in pot assays at 30 days **(B)**. The height of potato plants in pot assays at 30 days. **(C)** Representative photographs of potato roots inoculated with Y6 and control in field trials at 60 days. **(D)** The roots length and **(E)** the surface area of potato in field trials at 60 days.

SUPPLEMENTARY FIGURE S2 Effect of strain Y6 on the germination rate of potato tuber and the height of potato plant. **(A)** Potato tuber germination rate. **(B)** Potato plant height.

SUPPLEMENTARY FIGURE S3 Effect of strain Y6 on potato plant wet weight, yield per plant and plant stem thickness under field trials at 60 days. **(A)** Potato yield per plant. **(B)** Potato plant wet weight per plant.

## References

[ref1] Anand R. Grayston S. Chanway C. (2013). N2-fixation and seedling growth promotion of lodgepole pine by endophytic *Paenibacillus polymyxa*. Microb. Ecol. 66, 369–374. doi: 10.1007/s00248-013-0196-1, PMID: 23420205

[ref2] Balasubramanian V. Vashisht D. Cletus J. Sakthivel N. (2012). Plant beta-1,3-glucanases: their biological functions and transgenic expression against phytopathogenic fungi. Biotechnol. Lett. 34, 1983–1990. doi: 10.1007/s10529-012-1012-6, PMID: 22850791

[ref3] Batool T. Ali S. Seleiman M. F. Naveed N. H. Ali A. Ahmed K.. (2020). Plant growth promoting rhizobacteria alleviates drought stress in potato in response to suppressive oxidative stress and antioxidant enzymes activities. Sci. Rep. 10:16975. doi: 10.1038/s41598-020-73489-z, PMID: 33046721 PMC7550571

[ref4] Bottini R. Cassan F. Piccoli P. (2004). Gibberellin production by bacteria and its involvement in plant growth promotion and yield increase. Appl. Microbiol. Biotechnol. 65, 497–503. doi: 10.1007/s00253-004-1696-1, PMID: 15378292

[ref5] Braun S. Gevens A. Charkowski A. Allen C. Jansky S. (2017). Potato common scab: a review of the causal pathogens, management practices, varietal resistance screening methods, and host resistance. Am. J. Potato Res. 94, 283–296. doi: 10.1007/s12230-017-9575-3

[ref6] Cao Y. Pi H. Chandrangsu P. Li Y. Wang Y. Zhou H.. (2018). Antagonism of two plant-growth promoting *Bacillus velezensis* isolates against *Ralstonia solanacearum* and *Fusarium oxysporum*. Sci. Rep. 8:4360. doi: 10.1038/s41598-018-22782-z, PMID: 29531357 PMC5847583

[ref7] Dahal K. Li X. Q. Tai H. Creelman A. Bizimungu B. (2019). Improving potato stress tolerance and tuber yield under a climate change scenario—a current overview. Front. Plant Sci. 10:563. doi: 10.3389/fpls.2019.00563, PMID: 31139199 PMC6527881

[ref8] Di Salvo L. P. Cellucci G. C. Carlino M. E. García De Salamone I. E. (2018). Plant growth-promoting rhizobacteria inoculation and nitrogen fertilization increase maize (*Zea mays* L.) grain yield and modified rhizosphere microbial communities. Appl. Soil Ecol. 126, 113–120. doi: 10.1016/j.apsoil.2018.02.010

[ref9] Du M. M. Spalding E. P. Gray W. M. (2020). Rapid auxin-mediated cell expansion. Annu. Rev. Plant Biol. 71, 379–402. doi: 10.1146/annurev-arplant-073019-025907, PMID: 32131604 PMC7733314

[ref10] Etesami H. Maheshwari D. K. (2018). Use of plant growth promoting rhizobacteria (PGPRs) with multiple plant growth promoting traits in stress agriculture: action mechanisms and future prospects. Ecotoxicol. Environ. Saf. 156, 225–246. doi: 10.1016/j.ecoenv.2018.03.013, PMID: 29554608

[ref11] Feng R. Y. Chen Y. H. Lin C. Tsai C. H. Yang Y. L. Chen Y. L. (2022). Surfactin secreted by *Bacillus amyloliquefaciens* Ba01 is required to combat Streptomyces scabies causing potato common scab. Front. Plant Sci. 13:998707. doi: 10.3389/fpls.2022.998707, PMID: 36388520 PMC9664162

[ref12] Fries M. Ihrig J. Brocklehurst K. Shevchik V. E. Pickersgill R. W. (2007). Molecular basis of the activity of the phytopathogen pectin methylesterase. EMBO J. 26, 3879–3887. doi: 10.1038/sj.emboj.7601816, PMID: 17717531 PMC2000356

[ref13] Geng P. Zhang S. Liu J. Zhao C. Wu J. Cao Y.. (2020). MYB20, MYB42, MYB43, and MYB85 regulate phenylalanine and lignin biosynthesis during secondary cell wall formation. Plant Physiol. 182, 1272–1283. doi: 10.1104/pp.19.01070, PMID: 31871072 PMC7054866

[ref14] Jiang C. H. Yao X. F. Mi D. D. Li Z. J. Yang B. Y. Zheng Y.. (2019). Comparative transcriptome analysis reveals the biocontrol mechanism of *Bacillus velezensis* F21 against fusarium wilt on watermelon. Front. Microbiol. 10:652. doi: 10.3389/fmicb.2019.00652, PMID: 31001229 PMC6456681

[ref15] Kesten C. Menna A. Sanchez-Rodriguez C. (2017). Regulation of cellulose synthesis in response to stress. Curr. Opin. Plant Biol. 40, 106–113. doi: 10.1016/j.pbi.2017.08.010 28892802

[ref16] Lata H. Li X. C. Silva B. Moraes R. M. Halda-Alija L. (2006). Identification of IAA-producing endophytic bacteria from micro propagated *Echinacea* plants using 16S rRNA sequencing. Plant Cell Tissue Organ Cult. 85, 353–359. doi: 10.1007/s11240-006-9087-1

[ref17] Lin C. Tsai C. H. Chen P. Y. Wu C. Y. Chang Y. L. Yang Y. L.. (2018). Biological control of potato common scab by *Bacillus amyloliquefaciens* Ba01. PLoS One 13:e0196520. doi: 10.1371/journal.pone.0196520, PMID: 29698535 PMC5919641

[ref18] Liu K. Kang B. C. Jiang H. Moore S. L. Li H. Watkins C. B.. (2005). A GH3-like gene, CcGH3, isolated from *Capsicum chinense* L. fruit is regulated by auxin and ethylene. Plant Mol. Biol. 58, 447–464. doi: 10.1007/s11103-005-6505-4, PMID: 16021332

[ref19] Liu H. Wang J. Sun H. Han X. Peng Y. Liu J.. (2020). Transcriptome profiles reveal the growth-promoting mechanisms of *Paenibacillus polymyxa* YC0136 on tobacco (*Nicotiana tabacum* L.). Front. Microbiol. 11:584174. doi: 10.3389/fmicb.2020.584174, PMID: 33101258 PMC7546199

[ref20] Loria R. Kers J. Joshi M. (2006). Evolution of plant pathogenicity in Streptomyces. Annu. Rev. Phytopathol. 44, 469–487. doi: 10.1146/annurev.phyto.44.032905.091147 16719719

[ref21] Majeed A. Muhammad Z. (2020). An overview of the common bacterial diseases of potato in Pakistan, associated crop losses and control stratagems. J. Plant Pathol. 102, 3–10. doi: 10.1007/s42161-019-00362-y

[ref22] Maris A. Suslov D. Fry S. C. Verbelen J. P. Vissenberg K. (2009). Enzymic characterization of two recombinant xyloglucan endotransglucosylase/hydrolase (XTH) proteins of *Arabidopsis* and their effect on root growth and cell wall extension. J. Exp. Bot. 60, 3959–3972. doi: 10.1093/jxb/erp229, PMID: 19635745

[ref23] Puranik S. Sahu P. P. Srivastava P. S. Prasad M. (2012). NAC proteins: regulation and role in stress tolerance. Trends Plant Sci. 17, 369–381. doi: 10.1016/j.tplants.2012.02.004 22445067

[ref24] Roudier F. Fernandez A. G. Fujita M. Himmelspach R. Borner G. H. Schindelman G.. (2005). COBRA, an *Arabidopsis* extracellular glycosyl-phosphatidyl inositol-anchored protein, specifically controls highly anisotropic expansion through its involvement in cellulose microfibril orientation. Plant Cell 17, 1749–1763. doi: 10.1105/tpc.105.031732, PMID: 15849274 PMC1143074

[ref25] Ryu C. M. Farag M. A. Hu C. H. Reddy M. S. Kloepper J. W. Pare P. W. (2004). Bacterial volatiles induce systemic resistance in *Arabidopsis*. Plant Physiol. 134, 1017–1026. doi: 10.1104/pp.103.026583, PMID: 14976231 PMC389924

[ref26] Santoro M. V. Cappellari L. R. Giordano W. Banchio E. (2015). Plant growth-promoting effects of native *Pseudomonas* strains on *Mentha piperita* (peppermint): an in vitro study. Plant Biol. 17, 1218–1226. doi: 10.1111/plb.12351, PMID: 26012535

[ref27] Santos-Cervantes M. E. Felix-Gastelum R. Herrera-Rodríguez G. Espinoza-Mancillas M. G. Mora-Romero A. G. Leyva-López N. E. (2016). Characterization, pathogenicity and chemical control of *Streptomyces acidiscabies* associated to potato common scab. Am. J. Potato Res. 94, 14–25. doi: 10.1007/s12230-016-9541-5

[ref28] Son H. J. Park G. T. Cha M. S. Heo M. S. (2006). Solubilization of insoluble inorganic phosphates by a novel salt- and pH-tolerant *Pantoea agglomerans* R-42 isolated from soybean rhizosphere. Bioresour. Technol. 97, 204–210. doi: 10.1016/j.biortech.2005.02.021, PMID: 16171676

[ref29] Timmusk S. Nicander B. Granhall U. Tillberg E. (1999). Cytokinin production by *Paenibacillus polymyxa*. Soil Biol. Biochem. 31, 1847–1852. doi: 10.1016/s0038-0717(99)00113-3

[ref30] Vaghela B. Vashi R. Rajput K. Joshi R. (2022). Plant chitinases and their role in plant defense: a comprehensive review. Enzym. Microb. Technol. 159:110055. doi: 10.1016/j.enzmictec.2022.110055, PMID: 35537378

[ref31] Vukasinovic N. Wang Y. Vanhoutte I. Fendrych M. Guo B. Kvasnica M.. (2021). Local brassinosteroid biosynthesis enables optimal root growth. Nat. Plants 7, 619–632. doi: 10.1038/s41477-021-00917-x, PMID: 34007032

[ref32] Walker J. R. Ferrar P. H. (1998). Diphenol oxidases, enzyme-catalysed browning and plant disease resistance. Biotechnol. Genet. Eng. Rev. 15, 457–498. doi: 10.1080/02648725.1998.10647966, PMID: 9573613

[ref33] Wang J. Qu F. Liang J. Yang M. Hu X. (2022). *Bacillus velezensis* SX13 promoted cucumber growth and production by accelerating the absorption of nutrients and increasing plant photosynthetic metabolism. Sci. Hortic. 301:111151. doi: 10.1016/j.scienta.2022.111151

[ref34] Wanner L. A. Kirk W. W. Qu X. S. (2014). Field efficacy of nonpathogenic Streptomyces species against potato common scab. J. Appl. Microbiol. 116, 123–133. doi: 10.1111/jam.12336, PMID: 24034169

[ref35] Wei M. Zhang M. Huang G. Yuan Y. Fu C. Yu L. (2020). Coculture with two *Bacillus velezensis* strains enhances the growth of *Anoectochilus* plants via promoting nutrient assimilation and regulating rhizosphere microbial community. Ind. Crop. Prod. 154:112697. doi: 10.1016/j.indcrop.2020.112697

[ref36] Xu Y. X. Xiao M. Z. Liu Y. Fu J. L. He Y. Jiang D. A. (2017). The small auxin-up RNA OsSAUR45 affects auxin synthesis and transport in rice. Plant Mol. Biol. 94, 97–107. doi: 10.1007/s11103-017-0595-7, PMID: 28321650

[ref37] Yan Y. Xu W. Hu Y. Tian R. Wang Z. (2022). *Bacillus velezensis* YYC promotes tomato growth and induces resistance against bacterial wilt. Biol. Control 172:104977. doi: 10.1016/j.biocontrol.2022.104977

[ref38] Yin Y. Chen H. Hahn M. G. Mohnen D. Xu Y. (2010). Evolution and function of the plant cell wall synthesis-related glycosyltransferase family 8. Plant Physiol. 153, 1729–1746. doi: 10.1104/pp.110.154229, PMID: 20522722 PMC2923890

[ref39] Zhang T. Lv W. Zhang H. Ma L. Li P. Ge L.. (2018). Genome-wide analysis of the basic helix-loop-helix (bHLH) transcription factor family in maize. BMC Plant Biol. 18:235. doi: 10.1186/s12870-018-1441-z, PMID: 30326829 PMC6192367

[ref40] Zhang H. Xu F. Wu Y. Hu H.-H. Dai X.-F. (2017). Progress of potato staple food research and industry development in China. J. Integr. Agric. 16, 2924–2932. doi: 10.1016/s2095-3119(17)61736-2

[ref41] Zhou Y. Li Q. Peng Z. Zhang J. Li J. (2022). Biocontrol effect of *Bacillus subtilis* YPS-32 on potato common scab and its complete genome sequence analysis. J. Agric. Food Chem. 70, 5339–5348. doi: 10.1021/acs.jafc.2c00274, PMID: 35467346

