# Supplementary figures and images for: Biological control of potato common scab and growth promotion of potato by Bacillus velezensis Y6
Source: Front Microbiol. 2023 Dec 11;14:1295107. doi: 10.3389/fmicb.2023.1295107 (PMC10750399; doi:10.3389/fmicb.2023.1295107)

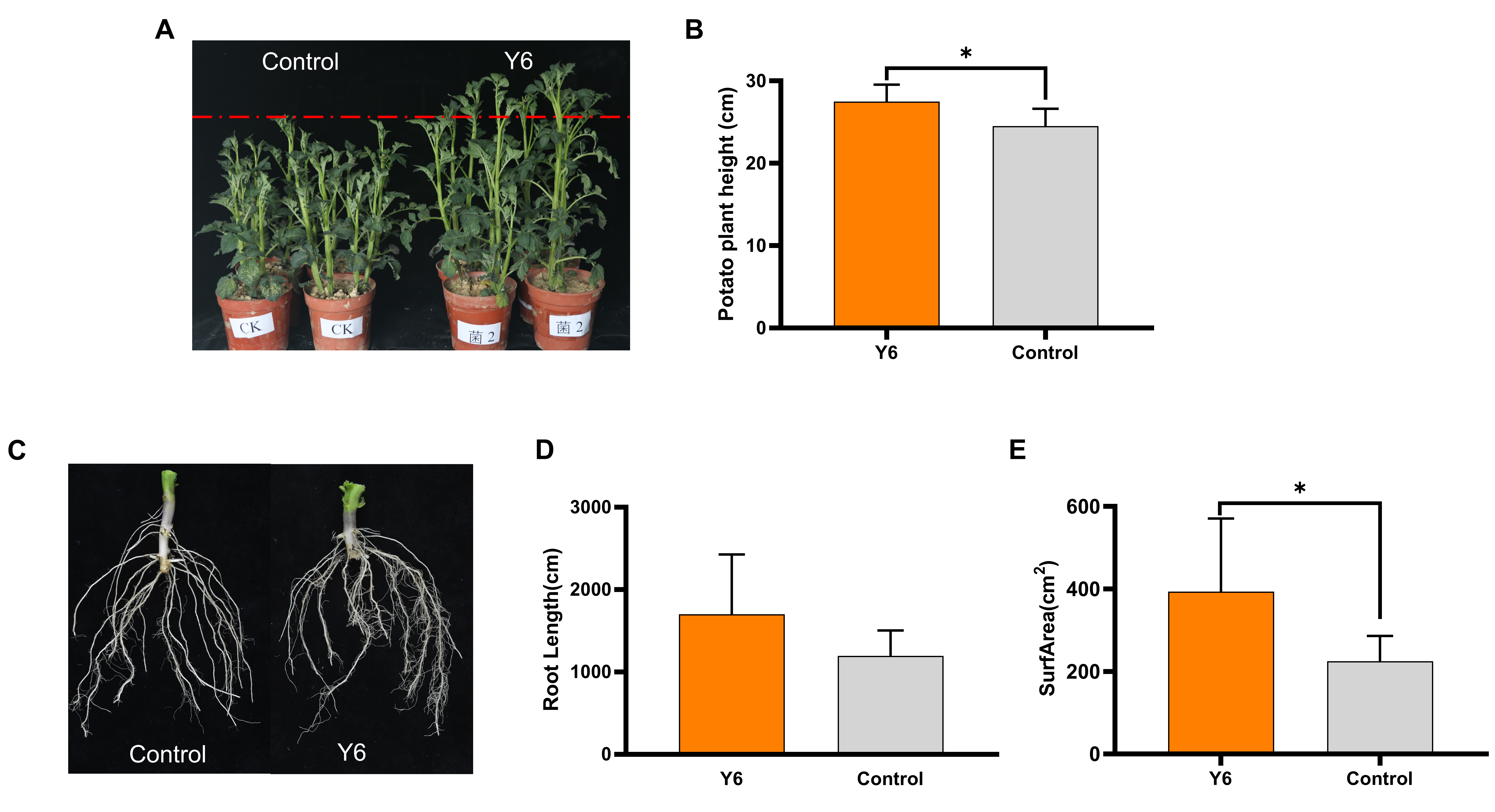

Supplement: SUPPLEMENTARY FIGURE S1 — Growth-promoting efficacy of Y6 on potato. (A) Representative photographs of potato plants to show the effects of the strain Y6 on the growth of potato in pot assays at 30 days (B). The height of potato plants in pot assays at 30 days. (C) Representative photographs of potato roots inoculated with Y6 and control in field trials at 60 days. (D) The roots length and (E) the surface area of potato in field trials at 60 days. [file Image_1.TIF]

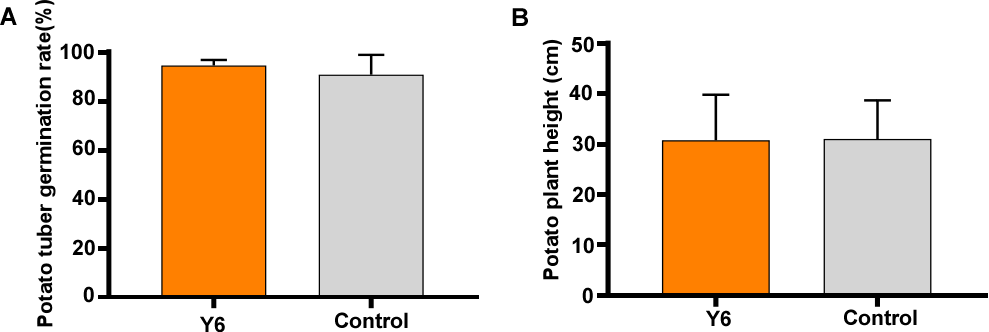

Supplement: SUPPLEMENTARY FIGURE S2 — Effect of strain Y6 on the germination rate of potato tuber and the height of potato plant. (A) Potato tuber germination rate. (B) Potato plant height. [file Image_2.TIF]

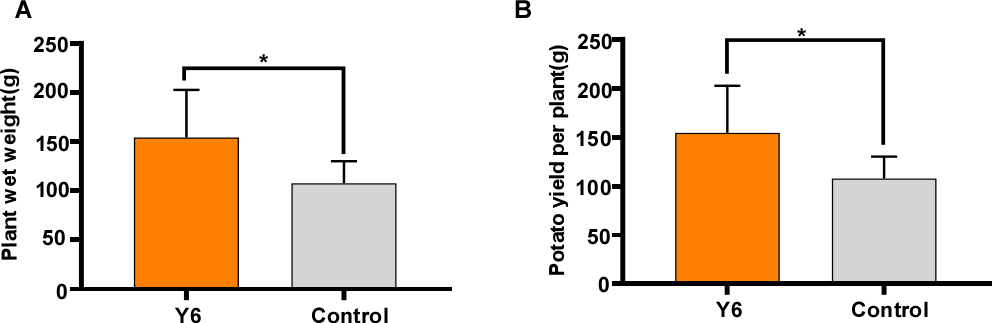

Supplement: SUPPLEMENTARY FIGURE S3 — Effect of strain Y6 on potato plant wet weight, yield per plant and plant stem thickness under field trials at 60 days. (A) Potato yield per plant. (B) Potato plant wet weight per plant. [file Image_3.TIF]
